# A Novel Electrochemical Sensor Based on Electropolymerized Ion Imprinted PoPD/ERGO Composite for Trace Cd(II) Determination in Water

**DOI:** 10.3390/s20041004

**Published:** 2020-02-13

**Authors:** Jiyang Wang, Jingfang Hu, Shiwei Hu, Guowei Gao, Yu Song

**Affiliations:** 1 Beijing key Laboratory of sensor, Beijing Information Science & Technology University, Beijing 100101, China; wang04252519@163.com (J.W.); hsw_0311@163.com (S.H.); ggw@bistu.edu.cn (G.G.); 2 Key Laboratory of Modern Measurement and Control Technology, Ministry of Education, Beijing Information Science and Technology University, Beijing 100192, China; 3 State Key Laboratories of Transducer Technology, Shanghai Institute of Microsystems and Information Technology, Chinese Academy of Sciences, Shanghai 200050, China

**Keywords:** cadmium determination in water, ion-imprinted polymer, PoPD/ERGO composite, electrochemical sensor

## Abstract

A novel electrochemical sensor based on electropolymerized ion imprinted poly (o-phenylenediamine) PoPD/electrochemical reduced graphene (ERGO) composite on glass carbon electrode (GCE) was fabricated for selective and sensitive determination of trace Cd(II) in water. ERGO was first deposited on the surface of GCE by electrochemical cyclic voltammetry (CV) scanning to enhance the electron transport activity at electrode surface. The ion imprinted polymer (IIP) of imprinted PoPD was then in situ electropolymerized on ERGO via CV scanning with oPD as functional monomer and Cd(II) ions as template, following removal of the template using electrochemical peroxidation method. The obtained imprinted PoPD/RERGO composites were characterized by scanning electron microscopy (SEM), transmission electron microscopy (TEM), and X-ray energy spectroscopy (EDS) for the observation of their morphologies and components. The electrochemical behavior of the imprinted PoPD/ERGO/GCE was performed by CV and SWASV. The fabricated sensor of the imprinted PoPD/ERGO/GCE showed a good selectivity toward target Cd(II) ions in the presence of other heavy metal ions. Under the optimized experimental conditions, the sensor exhibited a good linear relationship between SWASV stripping peak values and Cd(II) concentration in the range of 1 to 50 ng/mL, with the limit of detection as 0.13 ng/mL (S/N = 3). The proposed electrochemical sensor of imprinted PoPD/ERGO/GCE was successfully applied for trace Cd(II) determination in real water samples.

## 1. Introduction

Cd(II) as a heavy metal has been considered as one of the most harmful water pollutants due to its increasing usage in industrial production [1,2]. For human, Cd(II) can be easily ingested and accumulate in body through the food chain and damage the kidney, liver, bone, and blood, which may cause a huge threat to human health and a high destruction [3,4,5,6,7,8]. The World Health Organization (WHO) has defined the maximum permissible limit of concentration for Cd(II) as 3 ng/mL for Cd(II) in drinking water and the maximum allowable level for Cd(II) in surface water as 5 ng/mL because this level should not cause obvious health problems described above [9,10]. Therefore, the monitoring of Cd(II) pollution in water is necessary for the effective management of cadmium in the environment in order to protect human beings. Cadmium content in water, especially in surface water or drinking water is usually found at low or trace level; thus, analytical measurement methods with a high selectivity and a low limit of detection (LOD) are required.

Current available optical technologies for the determination of Cd(II) have been developed such as atomic fluorescence spectrometry (AFS) [11], atomic absorption spectrometry (AAS) [12], inductively coupled plasma mass spectrometry (ICP-MS) [13], and ultraviolet absorption spectrometry (UV) [14]. Although accurate results can be obtained, these measurement instruments are complicated, huge, expensive, time-consuming, and not suited for the urgent requirement of field application or real-time monitoring. Electrochemical methods, however, become more and more popular for Cd(II) determination because of their efficiency and reliability enhancement in simplicity, rapid response, sensitivity, and low cost for practical application [15,16,17,18,19]. Many of the electrochemical strategies are based on the modification of the working electrode with appropriate material that can enhance the electrocatalytic activity for Cd(II) redox on electrode surface, or with some particular reagent containing active groups that can have chelation interact with Cd(II) [20,21,22]. In the last decade, various inorganic materials or nanomaterials—such as gold nanoparticles [23], bismuth nanoparticle [24], bismuth-antimony film [25], multiwalled carbon nanotubes (MWCNTs) [26,27,28], and graphene-carbon nanotubes hybrid nanocomposite—have been prepared for electrode modification to accelerate electron transfer rate and improve the electrocatalytical reduction activity of the deposited cadmium during preconcentration step for the enhanced electrochemical signals of Cd(II) stripping peaks [29]. In addition, some organic reagents or modifiers such as carboxyl functionalized polypyrrole-graphene oxide modified Au electrode [30], ionophore–Nafion modified bismuth electrode [31], cupferron and amide N-functionalized cyclam derivatives modified carbon paste electrodes [32], Nafion-graphene nanocomposite modified GCE [33], polyfurfural/nanoporous silica modified GCE [14], have been investigated for efficient determination of Cd(II) because of their particular ligands on electrode surface toward target Cd(II) as the result of the chelation reaction between the ligands and Cd(II) ions. However, these modified electrodes did not provide a proper selectivity toward Cd(II) in the presence of some potential interferers like Hg(II), Cu(II), Ni(II), Mn(II), Zn(II), Fe(II), and Mg(II) for real sample analysis. It should also be mentioned that most of these modified electrodes require a strong acid aqueous environment as pH ≤ 2.0, which would not only cause secondary water pollution, but also result in trouble for direct monitoring in practical application. Therefore, there is still a real need for the development of a highly selective sensing material in the field of electrochemical sensors for trace Cd(II) determination.

Over the past decade, ion-imprinted polymer (IIP) based electrochemical sensor for monitoring heavy metal ion has become an attractive area of research [34]. IIPs offer artificial recognition sites, which are able to specifically rebind a target ion in the presence of similar ions and have been widely used in the field of water selective separation [35]. These IIP materials can be prepared by polymerization of functional and cross-linking monomers around a template target ion. After polymerization, the template ions are removed, and the polymer can provide binding or recognition sites with shape, size and functionalities similar to the target ion [36]. Therefore, compared with other materials, the superiority of IIPs is their reliable selectivity for the capture of target ion [37]. Electrochemical sensors with IIP as sensing element can combine outstanding characteristics, that the artificial ion recognition ability from IIP secures their high selectivity, while the electrochemical transduction offers a high sensitivity response in a short time and a small dimension size of the sensor. The electrochemical sensors with IIPs for Ni(II), Cu(II), Cr(III), Hg(II), As(III), and some other heavy metal ions have been reported [38,39,40]. However, most of the IIPs are based on conventional polymerization methods such as bulk polymerization, dispersion polymerization and precipitation polymerization. There are two main problems existed for these IIPs for electrochemical sensor development. On one hand, the preparation process is complicate and the functional monomers used in the conventional methods are always non-conductive that cause weak electrochemical signal; on the other hand, the thickness of these IIPs are difficult to be controlled with a thin-film for immobilization on working electrode surface. To solve these problems, some electroactive functional monomers were found to be able to imprint molecules or ions, and electropolymerization was applied for IIPs preparation [41]. IIPs prepared by electropolymerization are superior with respect to the film adherence to the working electrode surface and the controllable thin-film formation as well as simple and fast preparation process. More recently, several literatures have reported the formation of IIPs for electrochemical recognition of target heavy metal ions such as Cu(II) and Cr(VI) with a great improvement of the sensitivity and selectivity for real sample application [42,43], but there is few report about the determination of Cd(II) by using electropolymerized IIP on electrochemical transducer surface.

Poly(o-phenylenediamine) PoPD is an attractive conducting polymer used in electropolymerized imprinted polymers due to its interesting electrical and electrochemical behaviors [44]. It is worth mentioning that PoPD possess new multiple functionalities owing to the amine and secondary amino groups in the polymer chain, which can interact with heavy metal ions and has been widely used in adsorption of some heavy metal ions in water treatment [45]. To the best of our knowledge, there is no previous report on the preparation of Cd(II) imprinted PoPD for sensor development. Generally speaking, the detection sensitivity of the imprinted sensor is based on the amount of effective imprinted sites and electron-transport ability on the sensor surface. It is well known that graphene has drawn considerable research interest in the sensor field for its ultra-high electron transport rate, large specific surface area, excellent biocompatibility, good stability, and low cost [46,47]. Many studies have demonstrated that graphene acted an important role on improving the sensitivity for the imprinted sensors [48,49].

In this work, we report a novel electrochemical sensor based on electropolymerized ion-imprinted poly PoPD/electrochemical reduced graphene oxide (ERGO) composite on GCE for trace Cd(II) determination. Firstly, electrochemical reduced graphene oxide (ERGO) was electrodeposited on GCE surface (ERGO/GCE) via electrochemical reduction method in order to enhance the sensitivity of the sensor. Compared with CVD [50] and chemical reduction methods, electrochemical reduction method is considered the most simple and environmental-friendly way for graphene fabrication [51]. Then, Cd(II) imprinted PoPD polymer was in situ electropolymerized at ERGO/GCE surface with oPD (o-phenylenediamine) as functional monomer and Cd(II) as template ions. During electropolymerizing process, Cd(II) would bind with PoPD through chelation interaction between Cd(II) ions and amine and imine groups of PoPD. The template Cd(II) ions were thus embedded into PoPD and formed surface imprinted sites after template removal. The imprinted composite was characterized by scanning electron microscopy (SEM), transmission electron microscope (TEM), X-ray energy spectroscopy (EDS), and electrochemical methods of cyclic voltammetry (CV) and square-wave voltammetry (SWV). The prepared imprinted electrochemical sensor was successfully used to detect trace Cd(II) in water samples. The sensor performances of selectivity, repeatability, and stability were all investigated.

## 2. Experimental

### 2.1. Materials and Instrumentation

4 mg/mL graphene oxide (GO) solution was purchased from Fuzhou Yihuan Carbon Co. Ltd. (Fouzhou, China). Potassium ferricyanide (K_3_[Fe(CN)_6_]), potassium dichromate (K_2_CrO_4_), acetic acid (CH_3_COOH), Sodium acetate (CH_3_COONa) and Cadmium sulfate (CdSO_4_)were purchased from Sinopharm Chemical Reagent Co. Ltd. (Beijing, China). Sodium hydroxide (NaOH), potassium chloride (KCl), concentrated hydrochloric acid (HCl, 37%), magnesium sulfate heptahydrate (MgSO_4_·7H2O), manganese sulfate (MnSO_4_), ferric sulfate (Fe_2_(SO_4_)_3_), copper sulfate pentahydrate (CuSO_4_·5H2O), and nitric acid zinc (Zn(NO_3_)_2_) were purchased from Xilong Chemical Co. Ltd. (Guangxi, China). were purchased from Sinopharm Chemical Reagent Co. Ltd. (Beijing China). All chemicals were analytically pure. All solutions were prepared using deionized water.

All electrochemical experiments were performed using a Chenhua Instrument 660 Electrochemical analyzer CHI660E (Shanghai Chenhua Instruments, Shanghai, China). Ultrasonic cleaning equipment from Shenzhen Jielian Cleaning Co. Ltd., (Shenzhen, China). A typical three-electrode system consists of a GCE as working electrode, Ag/AgCl as a reference electrode and Pt as a counter electrode. Scanning electron microscope (SEM) analysis was got on SIGMA field emission scanning electron microscope made by Zeiss (Zeiss. Ltd., Beijing, China). Transmission electron microscope (TEM) analysis was done by on JEM-1200EX transmission electron microscope by Japan JEOL (JELO. Ltd., Qingdao, China). Energy dispersive X-ray spectroscopy (EDS) analysis was done by on JSM-7500F.

### 2.2. Fabrication of the Imprinted PoPD/ERGO/GCE

**Figure 1:**
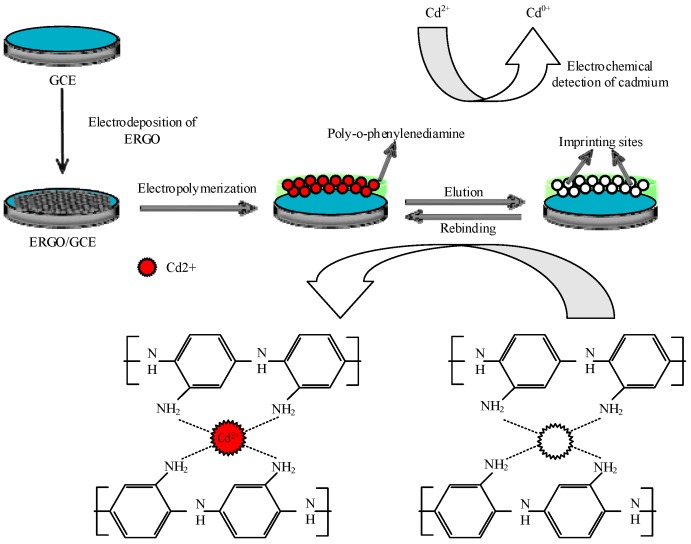
Detail procedure diagrams for fabrication of the IIP/ERGO/GCE.

The proposed sensor of the imprinted PoPD/ERGO/GCE was fabricated with two main steps (as shown in Figure 1). The first step is ERGO fabrication on GCE surface by the following process. A bare GCE and was carefully polished on the suede with Al_2_O_3_ powder until a mirror like surface was obtained, following with ultrasonic cleaning for 5 min in acetone, ethanol and deionized water, respectively. Prior to surface modification, the bare GCE was estimated in K_3_[Fe(CN)_6_] solution by cyclic voltammetry (CV) until a pair of well-defined redox peaks was observed, and the electrode was rinsed with deionized water and dried with nitrogen. After that, ERGO was electro-deposited on the GCE by cyclic voltammetry (CV) with the scan range of −1.5 V–1.0 V at 50 mV/s for 500 s in 0.1 M KCL supporting electrolyte containing 2.0 mg/mL GO. Then, the prepared ERGO/GCE was cleaned in deionized water 3 to 5 times to remove residual ERGO and KCl on the surface and dried with nitrogen. The second step is modification of IIP on ERGO using electropolymerization method. The ERGO/GCE was immersed in 0.1 M HAc-NaAc buffer solution (pH = 5.2) containing 0.04 M oPD as functional monomers and 0.01 M of Cd(II) as template ions. Then the IIP of Cd(II) imprinted PoPD was electropolymerized by CV scanning from 0.0 to 0.8 V at 50 mV/s for 20 cycles and template ions were removed by electrochemical peroxidation at 1.5 V for 15 min in 0.1 M HCl solution. As a comparison, the non-template imprinted PoPD (NIP/ERGO/GCE) was also prepared in the same condition except that no template ions were added in electrolyte during the electropolymerization stage.

### 2.3. Electrochemical Measurements

The prepared Cd(II)-imprinted PoPD/ERGO/GCE was immersed in a HAc-NaAc buffer solution (pH = 4.8) containing different concentrations of Cd(II). Square wave anode stripping voltammetry (SWASV) was used for the determination of Cd(II) under optimized conditions. First, Cd(II) ions were preconcentrated by electro-reduction of Cd(II) to Cd, which was deposited on the imprinted PoPD/ERGO/GCE surface at potential of −1.2 V for 300s under the stirred condition. Subsequently, the SWASV measurements were performed for Cd stripping in the potential range from −1.2 V to −0.4 V with a frequency of 15 Hz, amplitude of 25 mV, and a potential step of 4 mV. After each measurement, the electrode can be reusable by immersing in 0.1 M HCl at +1.5 V potential for 5 min under the stirred condition to remove Cd(II) ions adsorbed on the surface of the electrode. All measurements were performed at room temperature.

### 2.4. Real Water Sample Analysis

The prepared sensors were used to analyze real water samples collected from three different lakes and rivers around Beijing city. The water sample were stored in polyethylene bottles at 4 °C. It is worth noting that the pH of the water samples needs to be adjusted to 4.8. The Cd(II) in the real water sample was measured by SWASV, and the recovery rate was calculated to confirm the accuracy of the prepared sensor for practical use.

## 3. Results and Discussion

### 3.1. Preparation and Characterization of the Imprinted PoPD/ERGO/GCE

**Figure 2:**
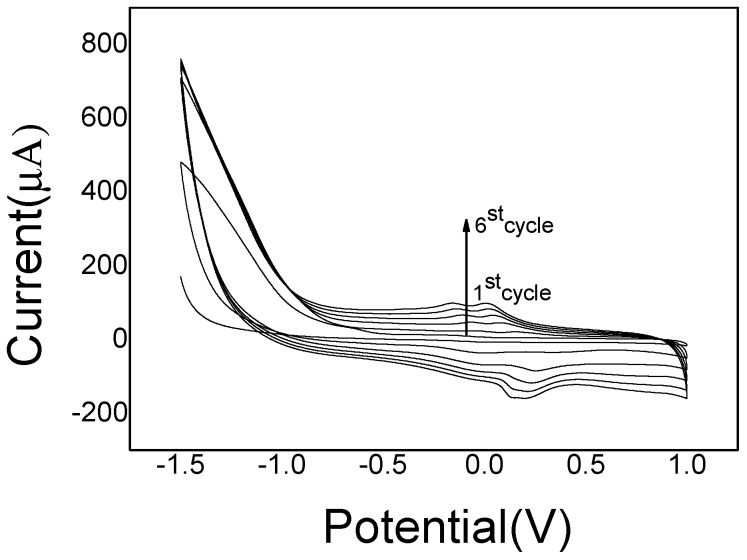
CV curves of electrodeposited ERGO on GCE.

**Figure 3:**
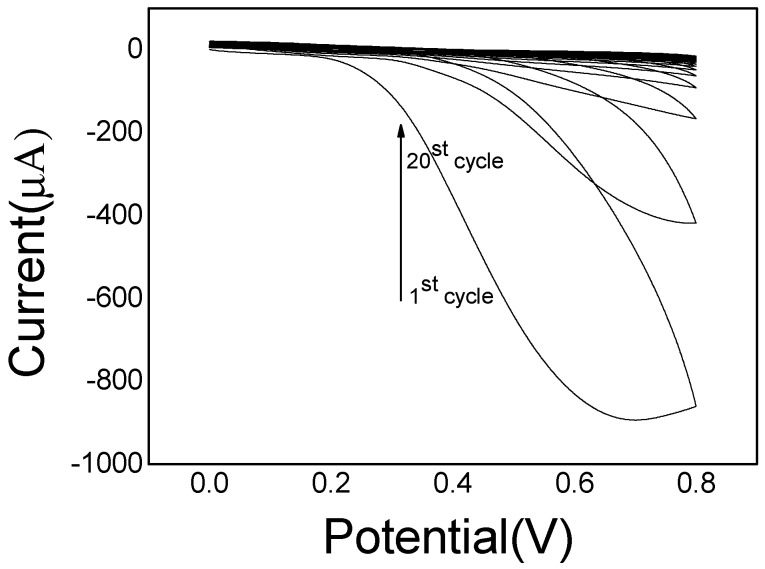
CV curve of electropolymerization of oPD on ERGO/GCE.

The proposed imprinted PoPD/ERGO composite was prepared with two steps. Firstly, the ERGO was electrodeposited on GCE surface by CV scanning in 2.0 mg/mL GO solution. As the CV shown in Figure 2, the current responses increased rapidly and reached a stable level with the increase of scanning cycles up to six cycles, suggesting that GO nanosheets were reduced and a stable ERGO layer formed on GCE surface. Subsequently, the imprinted PoPD film was electropolymerized on ERGO/GCE surface by CV scanning in 0.1 M HAc-NaAc buffer solution (pH = 5.2). As the CV electropolymerization process displayed in Figure 3, it was observed that an irreversible anodic oxidation peak appeared at the potential of 0.55 V. In the first scan, which may be ascribed to the oxidation of oPD monomer to its oxidized state [52].With the continual increase cycles, the anodic current gradually decreased and no obvious reduction peak was found during the electropolymerization, which revealed that electro-oxidation of oPD monomer occurred and a non-conductive layer was obtained on ERGO/GCE surface.

**Figure 4:**
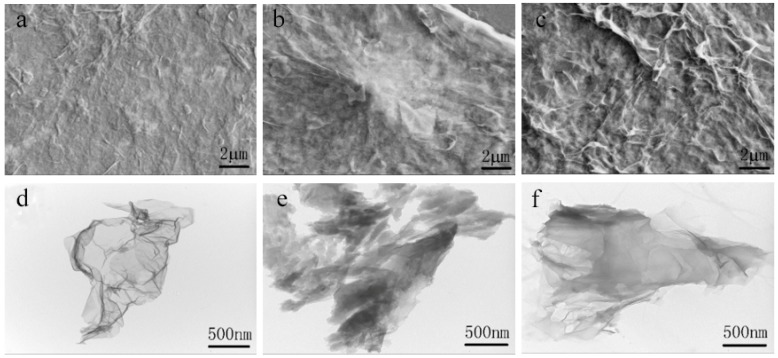
(**a**) SEM image of ERGO/GCE, (**b**) SEM image of Cd(Ⅱ)- IIP/ERGO/GCE, (**c**) SEM image of IIP/ERGO/GCE, (**d**) TEM image of ERGO/GCE, (**e**) TEM image of Cd(Ⅱ)-IIP/ERGO/GCE, (**f**) TEM image of IIP/ERGO/GCE.

**Figure 5:**
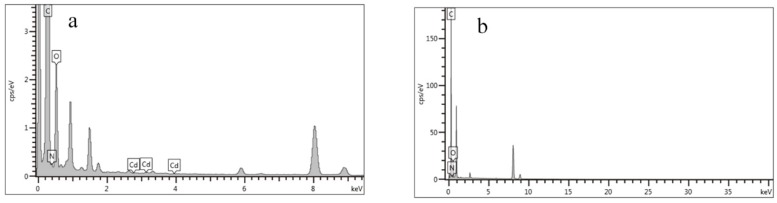
(**a**) EDS spectrum of Cd(II)-IIP/ERGO/GCE, (**b**) EDS spectrum of IIP/ERGO/GCE.

The surface morphology of the prepared ERGO/GCE, Cd(Ⅱ)-IIP/ERGO/GCE (containing Cd(II) in imprinted PoPD/ERGO/GCE before removal of template ions) and IIP/ERGO/GCE (after removal of template ions) were characterized by SEM and TEM, respectively. As shown the SEM image in Figure 4a and the TEM image in Figure 4d, it can be seen that the typical wrinkle and transparent structures of graphene were obtained on electrode surface, which indicated that ERGO nanosheets were successfully prepared on the electrode surface by electrochemical reduction method. Compared with the SEM image of Cd(II)-IIP/ERGO/GCE in Figure 4b, it can be observed that the IIP/ERGO/GCE in Figure 4c appeared rougher and more porous, which manifested that the template ions were successfully removed by electrochemical peroxidation method, and provided a larger surface area for the capture of target ions. The Cd(Ⅱ)-IIP/ERGO composite and IIP/ERGO composite were also compared by TEM characterization. As shown in Figure 4e,f, it is found that a thinner polymer layer was obtained on ERGO surface after template removal than the Cd(Ⅱ)-IIP/ERGO without removal of template. Additionally, EDS analysis of Cd(II)-IIP/ERGO/GCE and IIP/ERGO/GCE was further investigated in order to confirm the component difference as shown in Figure 5a,b. From which, Cd(II) spectrum peaks were found in Cd(II)-IIP/ERGO/GCE, but no Cd(II) spectrum peak was found in IIP/ERGO/GCE, suggesting that the template Cd(II) ions were washed off by electrochemical peroxidation method. The results demonstrated that the imprinted PoPD/ERGO composite was successfully prepared on GCE surface.

### 3.2. Optimization of the Conditions for IIP/ERGO/GCE Preparation

#### 3.2.1. Optimization of ERGO Deposition Time

**Figure 6:**
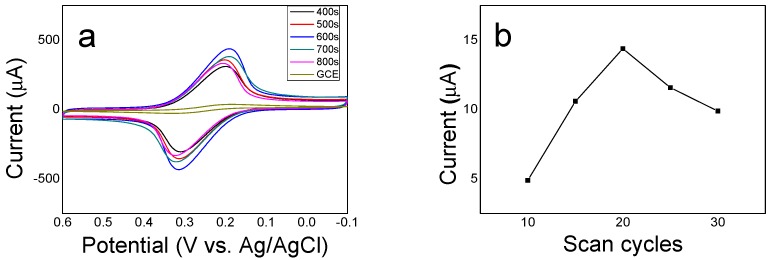
(**a**) CV curves of ERGO/GCE modified electrodes obtained with different deposition time in 0.1 M KCl containing 5 mM K_3_[Fe(CN)]_6_ solution, (**b**) Effect of the scanning cycles in electropolymerization process on the SWASV response of 10 ng/mL Cd(II) at a imprinted Cd(II)-IIP /ERGO/GCE.

ERGO nanosheets were electrodeposited on GCE by CV as signal amplifier material for the further IIP modification. The electrodeposition time is one of the important influence factors for the structure, density, and conductivity of the prepared ERGO on electrode surface, which has a strong correlation with the sensor sensitivity [53]. In this research, the deposition time of 400 s (4 cycles), 500 s (5 cycles), 600 s (6 cycles), 700 s (7 cycles), 800 s (8 cycles) were sequentially selected for ERGO electrochemical deposition using CV scanning in a potential range of −1.5 V‒1 V. Then, the prepared ERGO/GCE modified electrodes at different deposition time were examined respectively in 0.1 M KCl containing 5 mM K_3_[Fe(CN)]_6_. As shown in Figure 6a, it can be seen that the redox peak current increases with the increases of the deposition time from 400 s to 600 s, but, the peak current gradually decreases as the deposition time continually increases from 600 s to 800 s. The reason may be that the density of graphene nanosheets deposited on GCE surface gradually increased with the increase of deposition time, and the ERGO layer formed by strong π–π interaction, which can be able to effectively enlarge the specific surface area of electrode surface and improve of electronic transmission rate for sensitive. However, when the deposition time went up beyond a certain level, the stacked ERGO nanosheets could easily agglomerate together, which would hinder the electron transport and reduce electrocatalytical activity of the electrode surface and thus reduced the electrochemical responses [53,54,55]. Therefore, 600 s (6 cycles) was chosen as the optimum deposition time for ERGO preparation.

#### 3.2.2. Optimization of IIP Electropolymerization

The greatest advantage of the imprinted polymer prepared by eletropolymerization is its good controllability through adjustment of the electrochemical parameters such as potential, current or time, etc., [55]. In the case of sensor development, the thickness of sensing film is also an important factor affecting the sensor sensitivity [56]. When the imprinted sensing film is too thin, the imprinted sites would be not enough in the electropolymerized polymer, resulting a reduced sensitivity. However, if the imprinted films is too thick, the imprinted sites could be embedded too deep in the polymer, which will not only make it difficult to remove all the template ions, but also hinder the binding of target ions to imprinted sites [57]. Therefore, it is very necessary to prepare an ideal thickness of the imprinted PoPD film on ERGO/GCE via optimization of the electropolymerization time. Different CV scanning cycles were investigated for electropolymerization of Cd(II) imprinted PoPD. The prepared IIP/ERGO/GCE modified electrodes were examined respectively for their SWASV responses of 10 ng/mL Cd(II). The relationship between the stripping peak values and CV scanning cycles for electropolymerization of IIP was shown in Figure 6b, from which it was found that the SWASV peak response increased with the increase of scanning cycles and reached a maximum value when the number of scanning cycles is 20, but decreased as the scanning cycle number continuing to increase from 20 to 30. Therefore, 20 were chosen as the optimal number of CV scanning cycles for electropolymerization of the imprinted PoPD film on ERGO/GCE.

**Figure 7:**
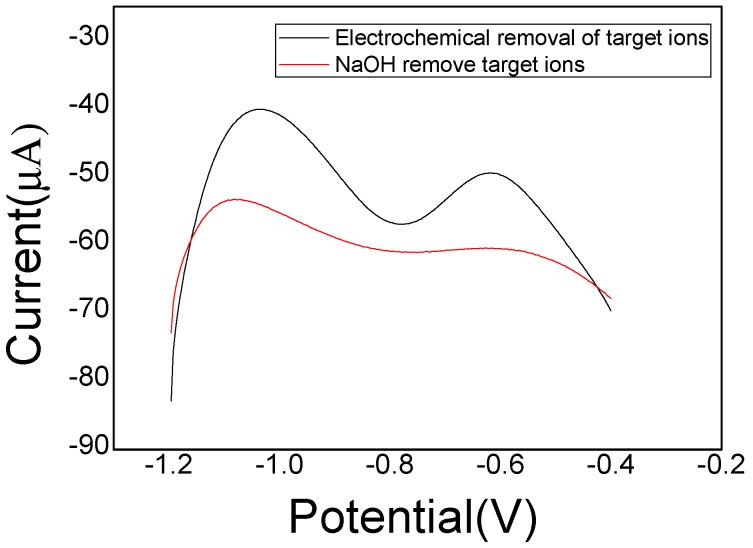
SWASV curves of different remove methods upon the 10 ng/mL Cd(II) standard solution.

As mentioned above, the amount of imprinted sites plays an important role on the sensitivity of the sensor, so it is really necessary to make sure that the embedded template Cd(II) ions should be removed completely. Two methods were compared between chemical washing solvent method and electrochemical peroxidation method were in order to obtain an optimal strategy to remove template ions from the IIP as completely as possible. The modified electrode from chemical washing solvent method (CS-IIP/ERGO/GCE) was prepared using NaOH to repeatedly wash the electrode surface for more than 1 h, and the other from electrochemical peroxidation method (EP-IIP/ERGO/GCE) was prepared using potentiostatic electrodeposition at +1.5 V for 15 min in 0.1 M HCl solution. As shown in Figure 7, it was seen that the SWASV stripping response at the EP-IIP/ERGO/GCE is obviously higher than CS-IIP/ERGO/GCE, suggesting that electrochemical peroxidation is more helpful to wash off the template ions embed in PoPD polymer. It is speculated that this phenomenon may be caused by the change of the conductivity of polymer matrix through the positive potential applied on the electrode, which limited the interaction between polymer film and template ions, generated electrostatic repulsion, thereby template ions are released from the imprinted PoPD polymer. Accordingly, electrochemical peroxidation was selected to remove the template ions for the final imprinted PoPD preparation.

### 3.3. Electrochemical Behavior of IIP/ERGO/GCE and NIP/ERGO/GCE

**Figure 8:**
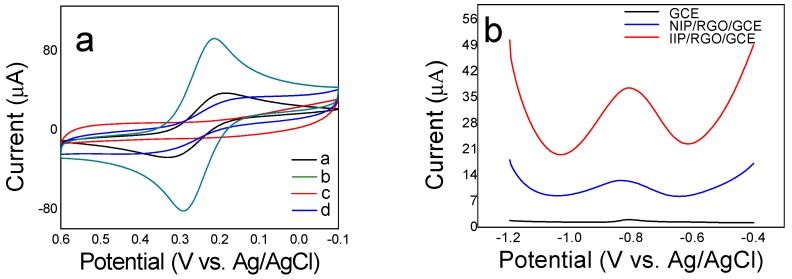
(**a**) CV curves on bare GCE (curve a), ERGO/GCE (curve b), Cd(II)-IIP/ERGO/GCE (curve c), and IIP/ERGO/GCE (curve d) between ‒0.2 V and 0.6 V at a scan rate of 50 mV/s in 5 mM K_3_[Fe (CN)]_6_ solution, (**b**) SWASV curves on bare GCE, NIP/ERGO/GCE and IIP/ERGO/GCE in 10 ng/mL Cd(II) standard solution.

The electrochemical behavior of different modified electrodes such as Cd(II)-IIP/ERGO/GCE, IIP/ERGO/GCE, and bare GCE was first studied in 0.1 M KCl containing 5 mM K_3_[Fe(CN)]_6_ by CV scanning. As shown in Figure 8a, it was found that a couple of typical redox peaks of K_3_[Fe(CN)]_6_ appeared at bare GCE (curve a) and enhanced redox peaks at ERGO/GCE (curve b), suggesting that the deposited ERGO improved the electron transport activity of the electrode surface. However, no peak and weak current responses were observed at Cd(II)-IIP/ERGO/GCE (curve c), which was though that the formed Cd(II) imprinted PoPD film by electropolymerization was very dense and compact, resulting that there would be almost no channels for the electron probe of [Fe (CN)_6_]^3−^ to approach the electrode surface. However, after removal of template Cd(II) ions, the IIP/ERGO/GCE showed an obvious increase of redox peak currents (curve d). This may attribute to the imprinted sites remained after removal of template, forming electron-transfer channels for [Fe(CN)_6_]^3−^ [58]. Then the electrochemical behavior of IIP/ERGO/GCE, NIP/ERGO/GCE, and bare GCE for Cd(II) sensing was investigated respectively using SWASV in 10 ng/mL Cd(II) solution (Figure 8b). The SWASV response at IIP/ERGO/GCE is significantly higher than bare GCE and NIP/ERGO/GCE, which suggested that the proposed imprinted PoPD/ERGO/GCE exhibited more sensitive response to recognize Cd(II).

### 3.4. Optimization of Analytical Conditions

#### 3.4.1. pH Optimization

**Figure 9:**
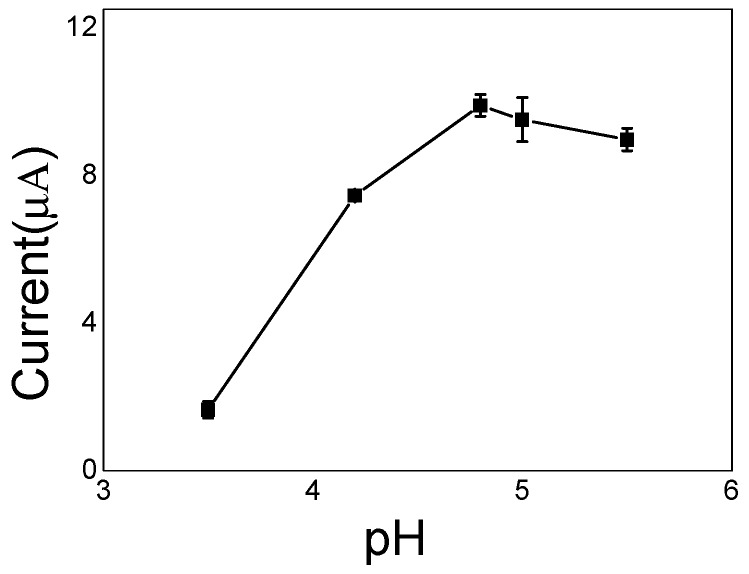
Relationship between stripping peak values recorded form SWASV responses and different pH in 10 ng/mL Cd(II) standard solution.

The presence of an acidic or basic functional group at the imprint sites on the IIP causes the adsorption and capture of the target ions to be greatly affected by the pH of the solution [59]. Different pH values from 3.5 to 5.5 were examined respectively for an optimum pH of water environment. As shown in Figure 9, the Cd stripping peak values were recorded from SWASV responses at IIP/ERGO/GCE in 10 ng/mL Cd(II) standard solutions with different pH from 3.5 to 5.5. It was found that stripping peak value increased along with the increase of pH from 3.5 to 4.8 and reached a maximum value with pH 4.8, and then decreased at a higher pH from 4.8 to 5.5. The possible reason was thought that at low pH (pH < 4.8), the functional groups on IIP/ERGO/GCE can be easily protonated, thereby reducing the chelating interaction between Cd(II) and IIP/ERGO/GCE [60,61]. Besides, when the pH is higher than 4.8 Cd(II) may combine with OH^+^ to form a precipitate, resulting in a decrease in electrode response [62]. Therefore, pH 4.8 was selected as the optimum pH value in this experiment.

#### 3.4.2. Optimization of Preconcentration Potential and Time

**Figure 10:**
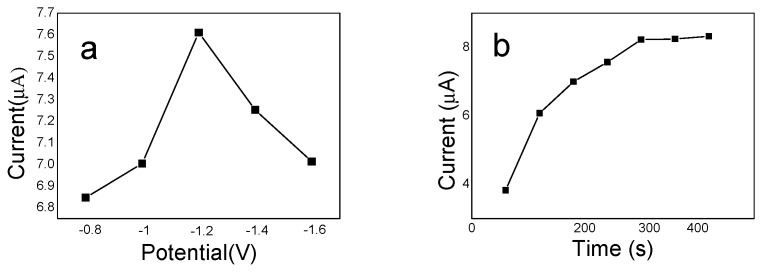
(**a**) Relationship between stripping peak values recorded form SWASV responses and preconcentration potential upon the 10 ng/mL Cd(II) standard solution, (**b**) Relationship between stripping peak values recorded form SWASV responses and preconcentration time upon the 10 ng/mL Cd(II) standard solution.

Preconcentration potential and time are very important for SWASV stripping of Cd on electrode surface that influences the sensor sensitivity and selectivity. The effect of preconcentration potential on the SWASV stripping peak response was studied in the range of −0.8 V to −1.6 V. As shown in Figure 10a. Cd(II) ions can be more easily reduced to Cd and deposited on electrode surface as the preconcentration potential shifts to the more negative direction in a certain range [29], that is why it showed that the stripping peak currents have an obvious increase in the range of −0.8 V to −1.2 V. While when the preconcentration potential became was more negative than −1.2V, the stripping peak current reduced greatly, may be due to hydrogen gas formation on the electrode surface and subsequent damage of the IIP/ERGO film [63]. Therefore, −1.2V was selected as the optimum preconcentration potential for the following measurement experiments. Furthermore, the effect of preconcentration time was also investigated by observation the relationship between the Cd SWASV stripping peak response and preconcentration time. Figure 10b showed that the stripping peak response increased with increasing the preconcentration time and reached a stable level at 300 s. This is because more and more Cd(II) would accumulated on the surface of the IIP/ERGO/GCE with increasing preconcentration time and reach resulting higher Cd stripping peak responses. A stable peak response obtained at 300 s may be owing to the Cd(II) ions accumulated on electrode surface up to a saturation state at 300 s. Therefore, 300 s was chosen as the optimum preconcentration time in this experiment.

### 3.5. Electrochemical Determination of Cd(II)

**Figure 11:**
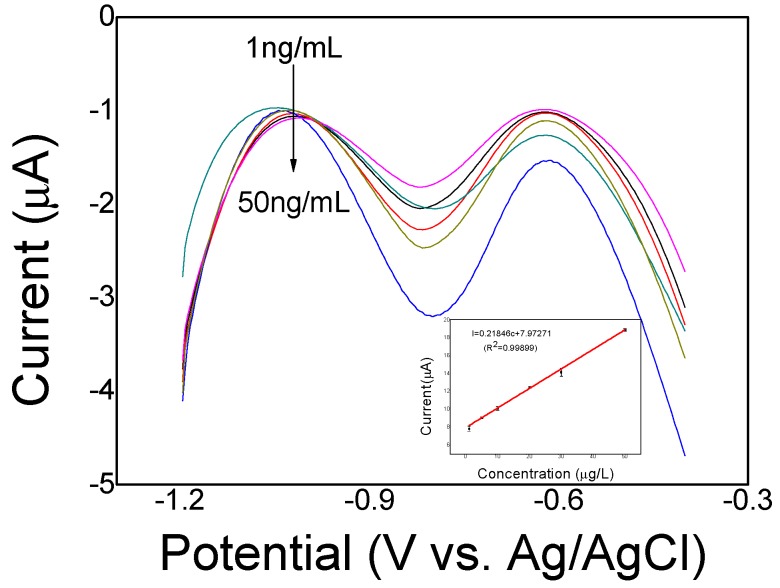
SWASV response curves at different concentrations. The inset shows the linear relationship between the current response and concentration from 1 ng/mL to 50 ng/mL.

The electrochemical determination of Cd(II) using the imprinted PoPD/ERGO/GCE was carried with SWASV in the optimized experimental conditions. As shown in Figure 11. It can be seen that there is a good linear relationship between the stripping peak current and the concentration of Cd(II) at a range of 1–50 ng/mL with a high sensitivity of 0.22 μA/ng/mL and correlation coefficient of 0.99899. The limit of detection was found to be 0.13 ng/mL according to S/N = 3. The sensing performances of linear range and LOD at the imprinted PoPD/ERGO/GCE were compared with those of other modified electrodes and the results are shown in Table 1. It is clear to see that proposed sensor offers a better linear range and detection limit than other reported Cd(II) sensor electrodes. It is almost the reason that the prepared IIP based electropolymerized PoPD has a stronger affinity to the target Cd(II) ions and the electrodeposited ERGO provides a great help in sensitivity improvement.

### 3.6. Selectivity, Repeatability, and Stability

**Figure 12:**
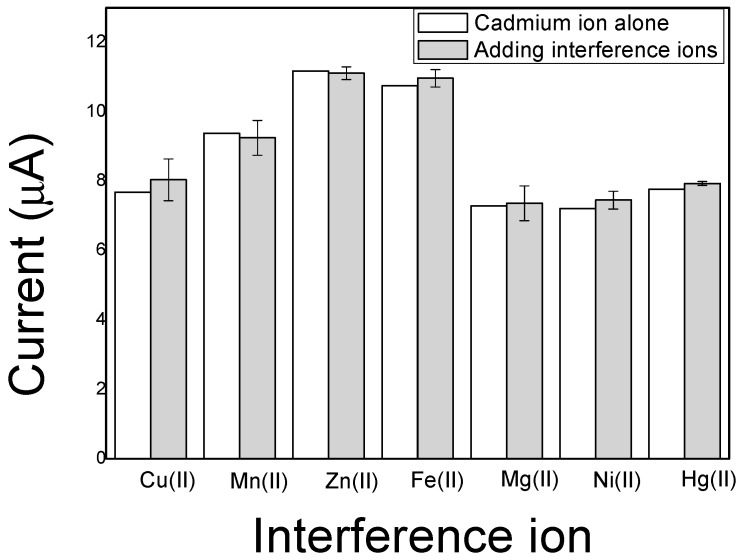
Effect of different interference ions on peak current detection of Cd(II).

In real application, the detection selectivity is always a great challenge from the other common heavy metal interference ions such as Hg(II), Ni(II), Cu(II), Mn(II), Zn(II), Fe(II), and Mg(II) for electrochemical sensing of Cd(II). Therefore, the SWASV responses of the imprinted PoPD/ERGO/GCE towards Cd(II) in the presence of the possible heavy metal ions of Hg(II), Ni(II), Cu(II), Mn(II), Zn(II), Fe(II), and Mg(II) were estimated respectively with a concentration of 20 times more than 10 ng/mL Cd(II) during measurement. In fact, the results in Figure 12 showed that the deviation is less than 5% of the Cd(II) response was observed in the presence of the other heavy metal ions, even if they concentration is 20 times than that of Cd(II), suggesting that these possible heavy metal ions have not significantly interference in the determination of Cd(II). The repeatability of the sensor was also studied by comparison of the SWASV stripping peak responses toward 10 ng/mL Cd(II) using five sensors were prepared in the same conditions. The measurement deviation of the five sensors is only 3.5%. Meanwhile, the stability of the sensor was evaluated by performing 10 repeated measurements in 10 ng/mL Cd(II) standard solution using the same sensor the maximum deviation of the SWASV peak current values was less than 10%. All the results revealed that the proposed electrochemical sensor of imprinted PoPD/ERGO/GCE is reliable for Cd(II) determination in water with good selectivity, repeatability, and stability.

### 3.7. Determination of Cd(II) in Real Water Samples

In order to evaluate the practicality of the proposed sensor, it was used for determination of Cd(II) in real water samples collected from lakes and rivers near Beijing city. No Cd(II) can be detected in these water samples. both using UV standard method and using the sensor. Therefore, the recovery test was conducted via the standard addition method using the known concentration of Cd(Ⅱ) to add in the water sample. The experimental results were presented in Table 2 and the recoveries were between 94% and 106.4%, indicating that the fabricated imprinted PoPD/ERGO/GCE sensor has a good recovery for practical application.

## 4. Conclusions

In this paper, an electrochemical sensor based on imprinted PoPD/ERGO composite was firstly reported and developed for trace Cd(II) determination in water. Two steps were conducted for the composite preparation, including direct electrodeposition of ERGO on GCE for amplification of electrochemical signal, and in situ electropolymerization of imprinted PoPD on ERGO for specific recognition of target Cd(II) ions. The imprinted PoPD/ERGO/GCE exhibited good selectivity and sensitivity, which the can be attributed to the plentiful and strong affinity binding sites on the porous IIP on ERGO toward target Cd(II) ions. In addition, a good repeatability and stability were also obtained. Moreover, the proposed electrochemical sensor was successfully employed for trace Cd(II) determination in real samples with good recoveries.

## Figures and Tables

**Table 1:** Comparison of the prepared sensor with other reported modified electrode for Cd(II) determination

Modified Electrode	Linear Range (ng/mL)	Detection Limit (ng/mL)	Ref.
CPE modified with cross-linked chitosan	6.63–168.6	1.1	[64]
GCE modified with stannum film	10–110	1.1	[65]
A bismuth-modified carbon nanotube electrode	2–100	0.7	[63]
Bismuth-powder modified carbon paste electrode	10–100	1.2	[66]
GCE modified with IIP/rGO nanocomposite	1–50	0.13	This work

**Table 2:** Determination of Cd(II) in natural water samples

Natural Water Sample	Cd(II) Added (ng/mL)	Found (ng/mL)	Recovery (%)
Sample 1	1.00	0.94	94
Sample 2	5.00	5.32	106.4
Sample 3	20.00	19.68	98.4
